# Digging Deep into Alzheimer Disease: How Electron Microscopy Helps Elucidating Its Pathogenesis

**DOI:** 10.1007/s10571-026-01676-z

**Published:** 2026-02-20

**Authors:** Sveva Dallere, Antonio Migheli, Alessandro Vercelli

**Affiliations:** 1 Department of Neuroscience “Rita Levi Montalcini”, Neuroscience Institute Cavalieri Ottolenghi, University of Turin, Regione Gonzole 10, 10043 Orbassano, Turin, Italy; 2 S.C. Neurologia 1, D.O.M.P. Centro Regionale Diagnosi Osservazione Malattie da Prioni, ASL TO2 Ospedale Maria Vittoria, Turin, Italy; 3 University School for Advanced Studies IUSS Pavia, Pavia, Italy

**Keywords:** Alzheimer disease, Electron microscopy, Beta amyloid, Hyperphosphorylated tau, Neuropathology, Diagnostics

## Abstract

Alzheimer disease (AD), first described by Alzheimer and Perusini in the early twentieth century, is a devastating neurodegenerative disorder without a definitive cure. Unraveling the subcellular alterations underlying AD is essential to elucidate disease mechanisms, track progression, link cellular abnormalities to functional deficits, and develop therapeutic strategies aimed at preventing, slowing or reverting the disease course. Electron microscopy (EM) has been pivotal in this field since the 1960s, when Terry and Kidd characterized the ultrastructure of amyloid-beta (Aβ) deposits and paired helical filaments (PHFs) composed of hyperphosphorylated tau. Over the decades, conventional transmission and scanning EM have been complemented by advanced approaches such as volume EM (vEM), cryo-electron microscopy (cryo-EM), and cryo-electron tomography (cryo-ET). These techniques enable three-dimensional reconstructions, minimize fixation artifacts, and provide near-native, near-atomic resolution insights into AD pathology. EM has also revealed critical contributions of other subcellular compartments to AD pathogenesis, including synapses, mitochondria, lysosomes, the blood–brain barrier, iron deposits, and inflammatory machinery. Importantly, EM studies extend beyond human tissue, encompassing animal models, cell cultures, and synthetic assemblies, thereby allowing cross-system comparisons that highlight conserved pathological features. By integrating data from diverse experimental settings, EM provides a uniquely comprehensive view of the AD subcellular landscape. This makes it an indispensable tool not only for dissecting disease mechanisms but also for guiding the rational design of therapeutic molecules with potential disease-modifying effects. This review synthesizes the state of knowledge on EM-based studies of AD, emphasizing their central role in advancing both mechanistic understanding and translational approaches.

**Graphical Abstract:**

Overview of electron microscopy approaches in AD research. Various experimental models, including human brain tissue, cell cultures, and animal models, are analyzed using TEM, SEM, cryo-EM, CLEM and vEM. Subsequent segmentation and analyses allow investigation of neuropathological features in AD, including Aβ plaques, Tau aggregates, synaptic alterations, mitochondrial dysfunction, neuroinflammation, autophagy deficits, BBB disruption, and iron accumulation. This image was created with BioRender.

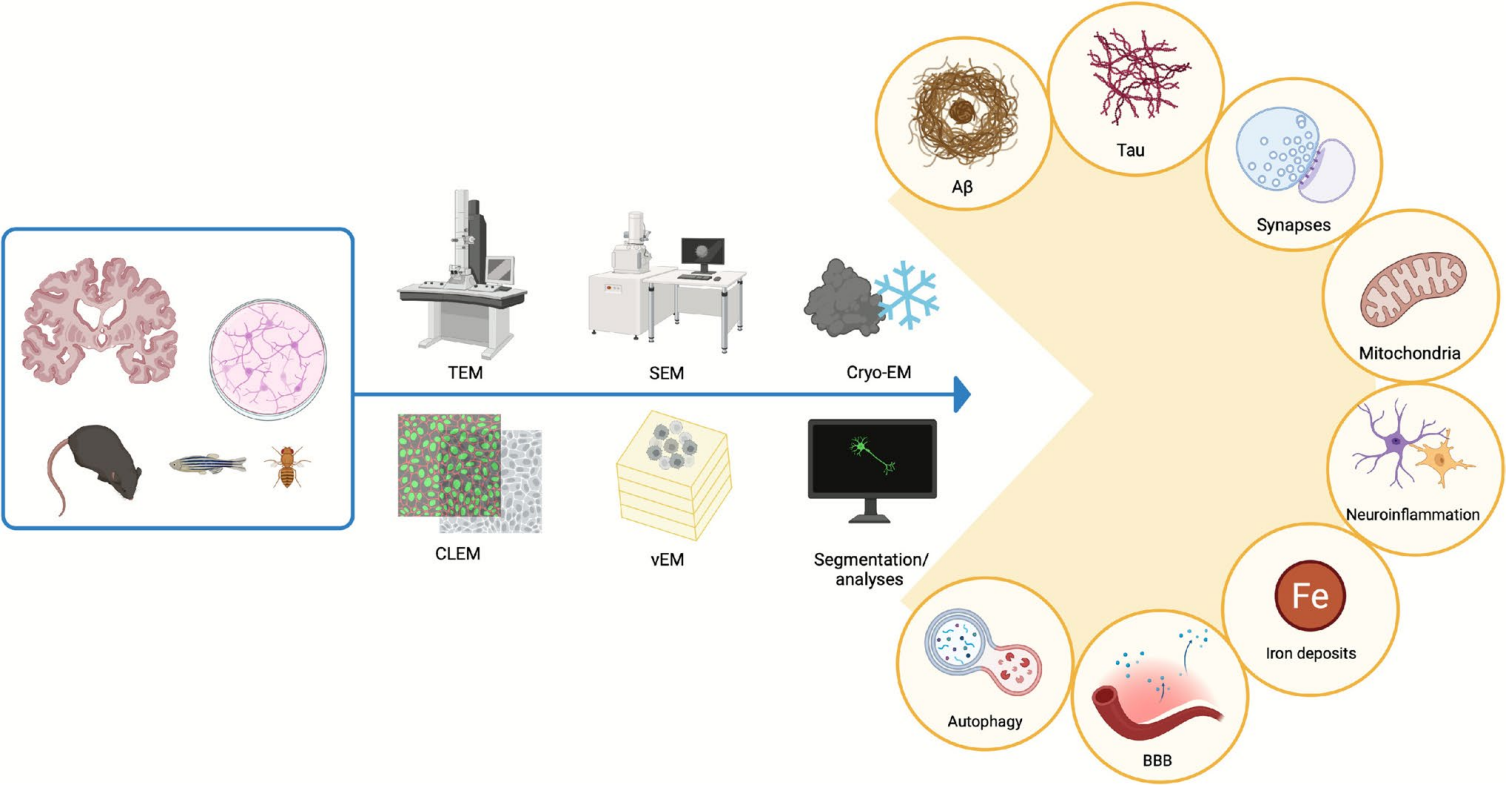

## Introduction

### Alzheimer Disease

Alzheimer disease (AD) is a progressive neurodegenerative disorder and the leading cause of dementia worldwide. Rather than arising from a single cause, its clinical and pathological features reflect a combination of genetic, lifestyle, and environmental influences, resulting in a complex network of molecular and cellular mechanisms. Classical hallmarks, including extracellular amyloid-beta (Aβ) plaques and intracellular neurofibrillary tangles (NFTs) composed of hyperphosphorylated tau (Selkoe and Hardy 2016), are accompanied by widespread synaptic dysfunction (Sheng et al. 2012), chronic neuroinflammation (Heneka et al. 2015), disrupted proteostasis (Cozachenco et al. 2023), and cerebrovascular changes (Sweeney et al. 2018a). Genetic risk factors, such as APOE ε4, further underscore the heterogeneity of its pathogenesis, in particular for sporadic AD (SAD) (Liu et al. 2013).

Given the complexity underlying the clinico-pathological picture of AD, understanding its ultrastructural correlates is essential to fully capture the pathobiological landscape of AD, as well as to provide novel findings which may inform the development of much needed therapeutic strategies aimed at slowing or reversing the clinical course.

### From Traditional to the Newest Technologies

Electron microscopy (EM) techniques have been instrumental in advancing our early understanding of the ultrastructural pathology associated with AD, offering detailed, high-resolution visualization of Aβ deposits and tau neurofibrillary tangles.

Starting from the seminal work of Terry et al. in 1964, traditional transmission electron microscopy (TEM) and scanning electron microscopy (SEM) have also served as key tools for investigating the density and organization of synapses in AD brains. These techniques have revealed region-specific mitochondrial accumulation and synaptic loss, particularly within hippocampal areas such as CA1 (DeKosky and Scheff 1990; Montero-Crespo et al. 2021), thus offering definitive proof of the morphological correlate of memory loss, one of the first clinical changes in AD.

Traditional methods provide nanoscale insights but only allow a bidimensional analysis of samples, thus hampering the possibility of following subcellular structures across the entire sample thickness. Emerging volume EM (vEM) approaches, such as focused ion beam SEM (FIB-SEM) and serial block-face SEM (SBF-SEM), offer three-dimensional reconstruction of large tissue volumes at nanometer resolution, facilitating detailed analysis of synaptic connectivity and pathology in AD (Montero-Crespo et al. 2021) (See Table 1).

For instance, FIB-SEM has enabled 3D quantification of synaptic loss in the AD hippocampus, highlighting layer-specific vulnerabilities (Montero-Crespo et al. 2021). Complementing these techniques, cryo-electron microscopy (cryo-EM) has shown to preserve native structures by vitrifying samples, thus avoiding the fixation artifacts which typically occur with traditional EM (Creekmore et al. 2024a), and has revolutionized AD research by resolving the structure of tau filaments and Aβ fibrils extracted from patients' brains at a near-atomic level (view Tables 2 and 3).

Advanced cryo-EM variants like cryo-electron tomography (cryo-ET) have extended the power of cryo-EM techniques to in situ imaging of cellular aggregates, revealing interactions between tau assemblies and organelles in near-native states (Leistner et al. 2023; Gilbert et al. 2024).

On the other hand, vEM, which combines high spatial resolution and volumetric imaging, has enabled the detailed reconstruction of neural circuits, cellular morphology, and synaptic architecture both in physiological and pathological situations (Calì and Wang 2025).

Integrating classical EM, vEM, and cryo-EM enables a multimodal approach to AD mechanisms, possibly deepening our understanding of neurodegenerative disease pathogenesis (Creekmore et al. 2021) and ultimately aiding the development of therapies.

Connected to these aspects, the emergence of large-scale datasets—reaching petavoxel dimension—obtained through vEM, has been paralleled by the development of advanced tools for segmentation and volume reconstruction, both manual and automated, driven in part by progress in machine learning Yin et al. 2020). This approach has been recently used by Han et al., who applied automatic segmentation and supervised learning for plaque segmentation and synapse identification (Han et al. 2023).

### Advantages and Limitations of EM Techniques in AD Research

While the various EM technique have substantially contributed to our understanding of the central nervous system, they also present distinct advantages and limitations, as it has been recently reviewed by Lujàn R et al. (Luján et al. 2024).

Among them, TEM is the most widely applied and continues to be fundamental for exploring the ultrastructure of nervous tissue, enabling visualization of organelles, membrane networks, cytoskeletal elements, and neuronal interconnections. On the other hand, SEM has a lower resolution and provides an image of the sample surface. However, they are traditionally limited to two-dimensional imaging; moreover, biological sample preparation is time-consuming and chemical fixation, dehydration and embedding may introduce artifacts (Luján et al. 2024). To overcome this issue, the cryo-EM technique has been developed, in order to preserve biological specimens at near native condition, via their vitrification within a thin amorphous ice film. Samples are then observed on a low dose TEM. Isolated biomolecular complexes can be analyzed and even proteins inside the cell can now be imaged in situ by FIB-milling or cryo-sectioning (Murata and Wolf 2018).

While cryo-EM has been central in unraveling molecular structures relevant to proteostasis (Creekmore et al. 2021), providing key insights into Aβ and tau filaments (Tables 2 and 3), several limitations remain in its application to AD pathology. Despite its high-resolution capabilities, cryo-EM is inherently limited by a small field of view, which restricts its ability to capture large-scale morphological features relevant to AD, such as plaque architecture, neurofibrillary tangle distribution, or cellular- and tissue-level context. Consequently, cryo-EM is poorly suited for probing spatial relationships between pathological aggregates and surrounding cellular structures. In addition, the technique remains still relatively low throughput, owing to demanding sample preparation, data collection, and computationally intensive image processing (Nogales and Scheres 2015; Creekmore et al. 2021). These limitations highlight the need to integrate cryo-EM with complementary approaches, such as light microscopy or cryo-ET, to achieve a multiscale understanding of AD pathology.

A widely used variant, negative-staining TEM (NS-TEM), offers a rapid and accessible approach for examining the morphology of isolated protein or macromolecular complexes (such as amyloid fibrils and tau filaments). By contrasting the sample with heavy-metal stains, NS-TEM provides high-contrast images that allow structural characterization of filament assemblies. In the context of AD, this technique has been used in combination with cryo-EM in the study of Aβ and tau structures (Tables 2 and 3). Nevertheless, the staining and drying processes can distort native conformations, and the achievable resolution (typically ~ 20 Å) is insufficient for atomic-level interpretation (Burgess et al. 2004; Booth et al. 2011).

Another 2D technique, developed to study the distribution of integral membrane proteins, is sodium dodecyl sulfate-digested freeze-fracture replica labeling (SDS-FRL) (Fujimoto 1995). The main advantage of this technique is its high sensitivity in detecting membrane protein and allowing quantitative studies of different antigens (Kaufmann et al. 2021), though it is technically demanding and restricted to surface-accessible epitopes. In AD research this approach has been used to study neurotransmitter receptor distribution at synaptic sites (Martín-Belmonte et al. 2021, 2020b; Alfaro-Ruiz et al. 2022).

Finally, scanning TEM (STEM) can both measure the mass of single-protein complexes and take images of such complexes, offering applications in structural biology (Sousa and Leapman 2012), as it has been done by Goldsbury et al. (2011) and Zeng et al. (2021).

vEM techniques overcome the dimensional limitation of traditional EM by enabling 3D reconstruction of large tissue volumes at nanometer resolution, revealing network-level organization and layer-specific vulnerabilities. For decades the gold standard in this field has been serial section TEM. The images are later stacked, allowing for volume visualization and analyses. However, this procedure is extremely time consuming and demanding and keeping serial section uniformity is particularly difficult (Miranda et al. 2015). Automated methods have been developed to overcome these issues. In this technique SEM is commonly used and images are usually obtained using backscattered electrons, which results in images very similar to those obtained with TEM (Zhao et al. 2024; Luján et al. 2024). In SBF–SEM, an ultramicrotome with a diamond knife is incorporated into a SEM. A disadvantage is it being a disruptive technique, because in each cycle the block face is destroyed. Conversely, in automatic tape-collecting ultra-microtome (ATUM)-SEM the sections are collected and can be imaged multiple times (Baena et al. 2019). Moreover, it can be accompanied by a multibeam SEM, which can be particularly useful in connectomics research (Eberle and Zeidler 2018). Another vEM approach is to mill the surface sample, instead of cutting the whole block face: in the FIB-SEM a gallium ion beam mills the surface at the desired thickness, allowing for a better Z-axis resolution (Knott et al. 2008).

As outlined above and discussed in greater detail in the subsequent sections, the advent of vEM has enabled major advances in the study of AD-related mechanisms, including synaptic, mitochondrial, and autophagy-associated alterations.

Finally, Cryo-ET uses TEM to image the sample at different angles and allows sub-nanometer resolution, however the sample that should be only 100–400 nm thick. Thus, this technique is suitable for characterizing macromolecular complexes but does not allow larger volume reconstruction (Murata and Wolf 2018; Gilbert et al. 2024).

Moreover, all these vEM techniques generate a huge amount of data, hence data analysis becomes a critical factor.

Overall, AD is a complex disorder, whose pathogenesis and mechanism of disease onset and progression is still debated. Due to its complexity, any approach—be it molecular, biochemical, genetic, morphological etc. – will have its inherent limitations. Only their integration can achieve a holistic view of the pathological landmarks of the disease, at different scales of investigation.

### Issues Related to Tissue Preservation

In the following paragraphs, studies on both human autoptical/bioptical samples and laboratory models of AD will be described. However, while non-human studies usually are based on transcardiac perfusion with paraformaldehyde and/or glutaraldehyde, therefore providing ideal ultrastructural preservation (Turner et al. 2022), in the case of human tissues, immersion fixation remains the only viable option (Karlupia et al. 2023). In surgical biopsy procedures, minimizing the length of ischemia is critical—ideally, only a few minutes should elapse between tissue removal and the initiation of fixation (Karlupia et al. 2023). Nonetheless, brain biopsies are typically obtained only in clinical scenarios that require surgical intervention (Kay et al. 2013). Therefore, in the AD field, human tissue is commonly derived from post-mortem autoptic brains, which even in the most desirable situation are fixed after 4 h post-mortem (Montero-Crespo et al. 2021).

Recently, cryofixation (vitrification) protocols have been developed to avoid chemical fixation artifacts and better preserve brain tissue ultrastructure in a state closer to its native condition (Creekmore et al. 2024a), while also improving ultrastructural preservation after long post-mortem intervals (Sele et al. 2019).

In this review, we have systematically examined the key hallmark and histopathological features of Alzheimer disease, emphasizing the pivotal role of electron microscopy in uncovering their ultrastructural characteristics. Currently available reviews have been usually focused on single aspects of the pathology (among them: Crowther and Goedert 2000; Hernández et al. 2005; Goldsbury et al. 2011; Lippens and Gigant 2019; Koike and Katsuno 2021; Crowther 2021).

Thanks to the inclusion of studies on human brain tissues as well as animal and cellular models, we have addressed the current gap in the literature by integrating both early foundational work and recent advances. This comprehensive overview offers a valuable resource on the diverse aspects of AD that can be explored through EM.

## Search Strategy and Selection Criteria

A comprehensive literature search was performed to identify original research articles investigating AD by means of EM. A search was conducted in PubMed which focused on studies examining the ultrastructural features of AD-related hallmarks such as Aβ, tau pathology, iron accumulation, neuroinflammation, BBB alterations, mitochondrial changes, synaptic integrity and autophagy.

The primary search string used in PubMed was as follows:

(“Alzheimer Disease”[MeSH Terms] OR “Alzheimer’s diseas”[Title/Abstract]).

AND (“amyloid bet”[Title/Abstract] OR “Aβ”[Title/Abstract]).

AND (“electron microscop”[MeSH Terms] OR “electron microscop”[Title/Abstract]).

AND (“result”[Title/Abstract] OR "experiment"[Title/Abstract] OR "data"[Title/Abstract]).

NOT (Review[Publication Type] OR Editorial[Publication Type] OR Case Reports[Publication Type] OR Letter[Publication Type]).

Similar queries were constructed for other topics of interest, by replacing the “amyloid beta” term with: “tau” OR “tau protein”**;** “iron” OR “metal accumulation”; “neuroinflammation”**;** “blood–brain barrier” OR “BBB”**;** “mitochondria” OR “mitochondrial”**;** “synapses” OR “synaptic”**;** “autophagy”.

The search was limited to articles published in English and included papers published in the last ten years. Additionally, for each topic, the very first papers in which EM was used to investigate the specific hallmark of AD were included. These results were then manually screened and studies with irrelevant titles and abstracts were excluded. In each paragraph, these works were included in the “Recent advances” and “Background”, respectively (See Table 1).

**Table 1:** The table shows the number of records identified on Pubmed for each AD hallmark, through the specific research string, the number of records published in the last 10 years and the number of papers included in the review, based on above mentioned inclusion/exclusion criteria

Hallmar	Records identified through database searching	Records in the last 10 years	Papers included
A $\beta$	619	393	39
Tau	175	118	35
Synapses	179	133	24
Mitochondria	104	84	25
Iron	70	38	16
BBB	181	55	16
Autophagy	85	81	20
Neuroinflammation	323	145	18

Inclusion criteria were:

- Original research articles presenting experimental data.
- Studies involving AD with a mechanistic or pathological focus.
- Research utilizing EM as a primary imaging modality.
- Articles reporting structural, ultrastructural, or subcellular findings relevant to AD pathology.
- Publications in English and peer-reviewed journals.

Exclusion criteria were:

- Reviews, editorials, letters, and case reports.
- Studies not involving EM as a primary imaging modality.
- Articles not related to AD or without a mechanistic/pathological focus.

## Main Text

### Amyloid

#### Background

The amyloid cascade hypothesis of Alzheimer disease suggests that a pathological event is the buildup of Aβ peptides within the brain (Selkoe & Hardy 2016). This theory is largely supported by neuropathological findings and human genetic evidence, particularly from familial cases of AD (FAD), while it is less applicable to sporadic AD (SAD) (Frisoni et al. 2022). Moreover, new Aβ-targeting drugs, such as Lecanemab, were successful in reducing amyloid burden, but they had moderate effect in slowing cognitive decline in early AD, in some cases at the expense of severe adverse effects, in clinical trials (Van Dyck et al. 2023).

In this context, a more accurate knowledge of the timing of Aβ deposition, of the behavior of different Aβ fragments and of their interplay with other AD hallmarks (tau pathology, neuroinflammation, synaptic and mitochondrial dysfunction etc.) is critical.

Senile plaques were first described in 1906 by Alois Alzheimer, and in full detail by his mentee Gaetano Perusini in 1909 (Rosso and Chu 2021), but only with the spread of EM, starting from the ‘60 s, their composition was clarified. Terry et al. (1964) (Terry et al. 1964) described for the first time the fibrillary core of plaques in cerebral biopsies of three cases of early onset AD. The central core was described as a “stellate mass of interwoven fibers, each 70 to 90 Å wide”; fibrils were grouped into bundles, generally oriented toward the center of the plaque and devoid of membranes. It was clear that central fibrils and bundles were extracellular, while those in the plaque periphery were found in close relationship to nuclei and organelles. Meanwhile, Shirahama and Cohen were able to describe the general structure of all amyloid fibrils: they are made of 5 parallel protofibrils (25–35 Å), which are in turn made of 2 or 3 subprotofibrils helically arranged (10–15 Å) (Shirahama and Cohen 1967). In 1980, these structures were also described at the ultrastructural level in brain biopsies from AD patients (Narang 1980).

In 1984, amyloid twisted β-pleated sheet fibrils were then purified from AD brain (Glenner and Wong 1984) and their amino acid sequence was found to have no homology with other known proteins. In 1985, immunogold EM labeling was used to demonstrate in vitro fibril formation from two synthetic peptides homologous to β-amyloid fragments (Castaño et al. 1986) and in 1987 similar results were obtained through x-ray diffraction and EM (Kirschner et al. 1987), thus allowing the identification of the specific sequence responsible for AD-like fibril formation. Finally, in 1989 Halverson et al. showed that actually the sequence 34–42 is responsible for the formation of stable, insoluble β-structures (Halverson et al. 1990).

#### Recent Advances

Since the 2010s, advances in both conventional EM and cryo-EM have significantly enhanced our understanding of Aβ ultrastructure (view Table 2 for further studies). Notably, cryo-EM has enabled near-atomic level visualization of Aβ fibrils. In 2017, Gremer and colleagues reported the structure of recombinant Aβ42 fibril, revealing two twisted protofilaments, by employing cryo-EM at 4.0 Å resolution in combination with solid-state nuclear magnetic resonance techniques. They also resolved the Aβ42 backbone of 42 residues, including the entire N-terminus (Gremer et al. 2017).

This structure was later refined and complemented by subsequent studies (Ghosh et al. 2021), which uncovered further conformational polymorphisms across fibrils purified from patients’ meninges or cortex. Moreover, Yang et al. (Yang et al. 2022) reported that Aβ42 extracted from human brain tissue forms two types of filaments. Type I was predominant in SAD patients, whereas Type II was mainly observed in familial AD (FAD) cases and represented the dominant form in AppNL-F knock-in mice (Table 2).

These results underscore the high heterogeneity of amyloid fibrils and aggregates; indeed amyloid deposition was demonstrated to be different among diverse tissues (Yang et al. 2023b, a), in various mice models (Zielinski et al. 2023) and in recombinant Aβ fibrils that were formed in vitro (Kamalaldinezabadi et al. 2024; Zielinski et al. 2025) (Table 2).

**Table 2:** Cryo-EM contribution to the understanding of Aβ structure: this table summarizes key studies describing Aβ conformation, with the use of Cryo-EM alone or in combination with other techniques

Fragment/Residue	Conformation	Sample origin	Technique	Reference
Aβ42	The individual layers of the Aβ fibril are formed by peptide dimers with face-to-face packing. The two peptides forming the dimer possess identical tilde-shaped conformations and interact with each other by packing of their hydrophobic C-terminal β-strands	Synthetic Aβ42	Cryo-EM	Shmidt et al. (2015)
Aβ42	Two intertwined protofilaments. Backbone of all 42 residues, nearly all side chains and N terminus are resolved. "LS"-shaped topology of individual subunits	Recombinant Aβ42	Cryo-EM	Gremer et al. (2017)
Aβ40, Aβ38, Aβ(2–40), Aβ37, Aβ36, and Aβ39	Right-hand twisted fibrils; polymorphic, assuming three main different morphology	AD patient and control leptomeninges extracts	Cryo-EM	Kollmer et al. (2019)
Aβ40	Two fold screw symmetry about the fibril growth axis; β-hairpin conformations for molecules in the outer cross-β layers	Seeded fibril growth from AD patient cortical tissue extracts	Cryo-EM	Ghosh et al. (2021)
Aβ42	Type I predominant in FAD, type II predominant in SAD patients and *App*^NL−F^ knock-in mice, both left-handed	Human AD and *App*^NL−F^ knock-in mice brain extracts	Cryo-EM	Yang et al. (2022)
Aβ42	Type A and B fibrils adopted ν-shaped and υ-shaped conformation respectively, with opposite helical handedness	Seeded growth of second-generation brain-seeded Aβ42 fibrils, grown by incubating solutions of synthetic or recombinant Aβ42	NS-TEM, Cryo-EM	Lee et al. (2023)
Aβ plaques	Complex molecular architecture of β-amyloid plaques that includes fibrils, protofilament-like rods and branched amyloid	In situ in *App*^*NL−G−F*^ FAD mice	Cryo-CLEM, Cryo-ET	Leistner et al. (2023)
Aβ filaments containing the E22G Arctic mutation	Tetrameric filaments containing the E22G Arctic mutation differ from dimeric type I and type II filaments of wild-type Aβ42; they form additional hydrogen bonds. The majority of protofilaments are made of Aβ40, forming human Arctic fold A and B	Brain extract from a patient with APP Arctic mutation (case AβPParc1) and mouse knock-in line App^NL−G−F^	Cryo-EM	Yang et al. (2023a, b)
Aβ40 filaments	The ordered core of protofilaments comprises D1–G38. In each pair, H14–G37 pack against each other in an anti-parallel fashion. Aβ40 filaments from blood vessels are different from Aβ42 in plaques	Leptomeninges of AD and cerebral amyloid angiopathy patients	Cryo-EM	Yang et al. (2023a, b)
Aβ fibrils	Novel Aβ fibril structures were found in the APP/PS1, ARTE10 and tg-SwDI models, while the human type II filament fold was found in the ARTE10, tg-APP_Swe_ and APP23 models. Aβ fibril structure in tg-APP_Swe_ resemble the most type I fibrils of SAD patients	Seven different mouse model of AD	Cryo-EM	Zielinski et al. (2023)
Aβ40 fibrils in the presence of lipid vesicles	The dominant fibril is L1, composed of two intertwined protofilaments of the same fold, related by an approximate pseudo 2_1_ screw symmetry. It’s almost identical to Aβ fibrils from seeded fibril growth from AD patients	In vitro fibrillization of recombinant wild-type Aβ40	Cryo-EM	Frieg et al. (2024)
Aβ plaques	Plaques contain a mixture of fibrils, some of which are branched, and protofilaments, arranged in parallel arrays and lattice-like structures	In situ in human post-mortem AD brains	Cryo-CLEM, Cryo-ET, cryo-FIB-SEM	Gilbert et al. (2024)
Cotton wool plaques (CWP)	CWPs contain mainly type I filaments. They are present in two novel arrangements, type Ic and type Id, that may be responsible for the formation of CWPs	FAD patients with PSEN1 mutations brain extracts	Cryo-EM	Hoq et al. (2024)
150 kDa Aβ42 oligomers	150 kDa Aβ42 oligomers form higher-order string-like non-fibrillar assemblies	Synthetic Aβ42	NS-TEM, Cryo-EM	Kamalaldinezabadi et al. (2024)
Uppsala APP mutation (Δ690-695 in APP)	Two identical S-shaped protofilaments with an ordered fibril core of S8-A42. Synthetic polymorphs differ from the murine and human structure	Brain extract from tg-UppSwe mouse and from a patient with *Uppsala APP* mutation. In vitro fibril formation from four synthetic AβUpp(1–42)_Δ19–24_ monomers	Immunogold NS-TEM, Cryo-EM	Zielinski et al. (2025)

The table lists the type of fragment, aggregate or mutation studied; the features identified; whether the sample was synthetic, extracted from human or murine tissue or in situ; the electron microscopy technique employed and the corresponding reference. Altogether, these studies highlight the structural diversity of Aβ fibrils across AD and related pathologies, as well as between recombinant and brain/meninge-derived assemblies. The studies are reported in chronological order. (Schmidt et al. 2015; Yang et al. 2022, 2023b, 2023a; Lee et al. 2023; Leistner et al. 2023; Zielinski et al. 2023, 2025; Frieg et al. 2024; Hoq et al. 2024; Kamalaldinezabadi et al. 2024)

These studies further emphasize that investigating structural variations in Aβ fibrils may help future investigations linking Aβ profiling with severity, progression rate or clinical manifestations in AD (Yang et al. 2022). Understanding these variations is therefore crucial for developing inhibitors and imaging agents with both therapeutic and diagnostic potential (Ghosh et al. 2021).

Another striking result of the most recent ultrastructural studies is that the Arctic (E22G) mutation in amyloid precursor protein (APP) enhances Aβ40 fibril accumulation and cross-propagation in FAD, with higher Aβ40 deposition in plaque core compared to SAD (Tehrani et al. 2024).

**Fig.1:**
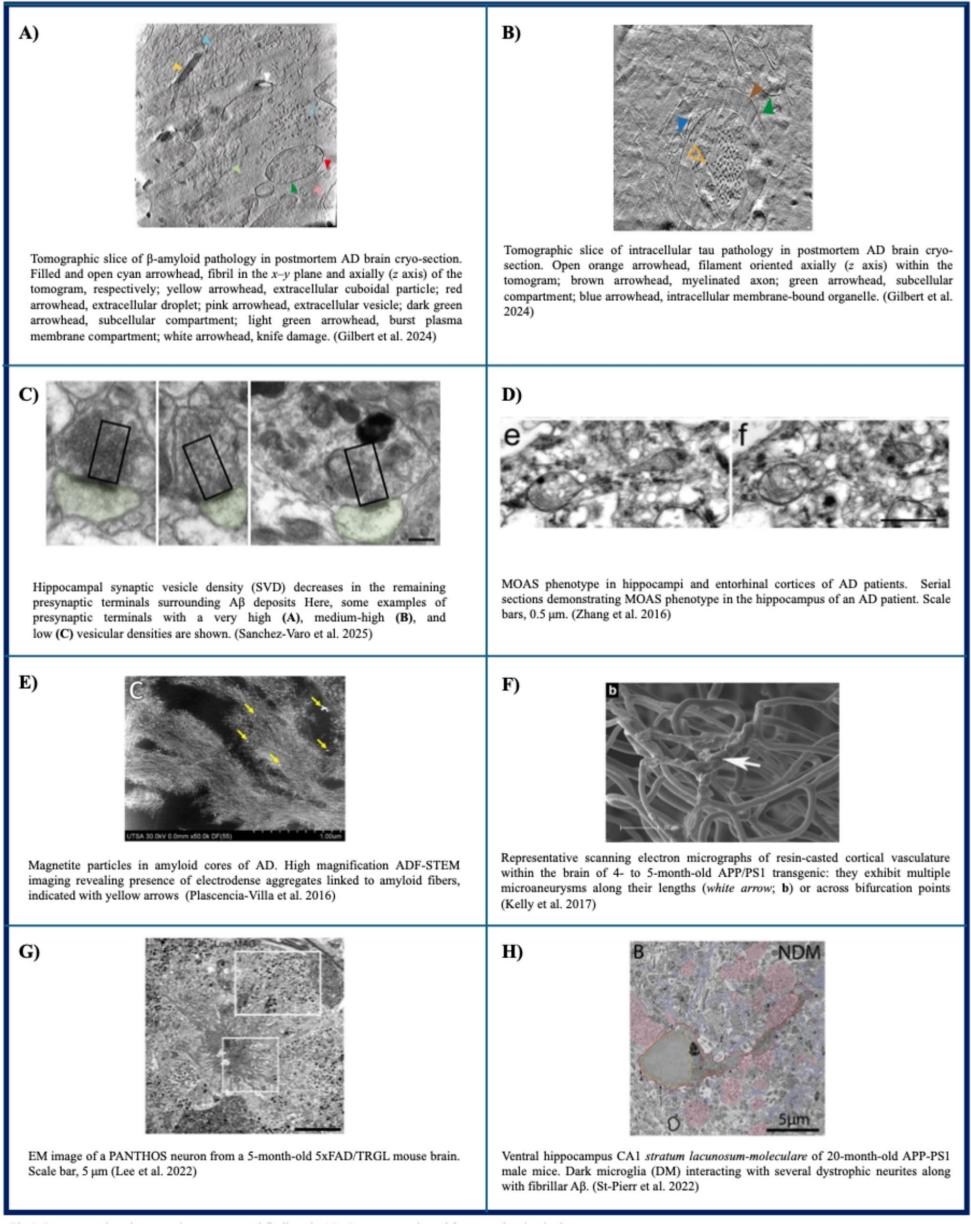
Representative EM novel findings in AD. Images are adapted from previously cited, open-source papers

Despite these observations, the in situ structure of amyloid in human brain remained elusive and was elucidated by Gilbert et al. only in 2024 (Gilbert et al. 2024) (Fig. 1A). Using cryo-correlative light-electron microscopy (CLEM) and cryo-ET, it was shown that Aβ plaques are composed of a heterogeneous mixture of fibrils—including branched forms—and protofilaments, organized in parallel arrays and lattice-like architectures. In addition, the plaques were found to be interwoven with various membrane-bound subcellular compartments, such as extracellular vesicles (EVs), lipid droplets and fragmented membranes.

Beyond allowing its structural characterization, EM has been pivotal in localizing Aβ at subcellular resolution. Indeed, recent investigations on thin sections of AD cerebral cortex found that intracellular Aβ α-sheet structures are intracellularly associated to lipofuscin, although pathobiological implication of this association are unknown. This raises new hypotheses regarding the formation of toxic oligomeric intermediates (Serwer et al. 2022) and shifting the focus from fibrils to soluble assemblies in disease initiation.

Besides these outstanding results, advanced immunogold EM techniques have shown that oligomeric Aβ species accumulate within synaptic terminals in a mouse model of AD, while array tomography has allowed the identification of different oligomeric species present in these subcellular structures (Pickett et al. 2016). Indeed, Aβ oligomers are known to interact with the neuronal membranes and induce perforations that cause an influx of calcium ions and enhance the release of synaptic vesicles, leading to delayed synaptic failure by vesicle depletion, as proved by TEM (Parodi et al. 2010).

Furthermore, the combination of different techniques, such as surface plasmon resonance and immunogold labeling, followed by direct analysis of the sensor-chip surface by SEM, has allowed to observe the lateral binding of Apolipoprotein E along the amyloid fibrils (Islam et al. 2019), thus opening the avenue for identifying novel potentially pathogenic interactions of Aβ.

In in vitro studies, the effect of Aβ on different cell types has also been elucidated via EM. For instance, Aβ was able to induce excessive mitochondrial fission in endothelial cells, leading to apoptosis (Zhang et al. 2025). Similarly, Aβ exposure was shown to induce pyroptotic features in cultured astrocytes (Hong et al. 2023).

Interestingly, recent EM-based investigations have also extended the study of Aβ beyond the traditional CNS tissue. Indeed, one challenge in neurodegenerative diseases is to identify peripheral biomarkers as minimally invasive diagnostic tools to be exploited at early or even preclinical stages of the disease. Notably, Aβ aggregates have been identified by immunogold EM in the cytoplasm of supranuclear fiber cells of the lenses in both human patients and murine models of AD and Down syndrome (Moncaster et al. 2010). In the same context, SEM and immunogold labeling have allowed to observe Aβ aggregates in periodontal biofilms (Kanagasingam et al. 2022) and in circulating neuron-derived extracellular EVs (Eitan et al. 2023).

Finally, EM has also been a useful tool to characterize alternative models of AD involving Aβ accumulation. For instance, in both APPL-RNAi and transgenic Drosophila melanogaster lines expressing eye tissue-specific human Aβ, eye degeneration was reported through SEM (Singh et al. 2017); while in a SAD-zebrafish model, TEM has highlighted the presence of higher order insoluble amyloid fibrils with twists (Dhiman et al. 2025).

Taken together, the evidence presented here highlights how EM has been fundamental in the initial characterization of senile plaques and how, in recent years, its progressive refinement has been pivotal in elucidating the ultrastructural complexity and localization of Aβ aggregates, as well as in characterizing novel models of Alzheimer disease—thereby significantly advancing our understanding of its pathogenesis.

### TAU

#### Background

The microtubule associated protein tau has been identified as the main component of the insoluble aggregates that characterize a number of neurodegenerative diseases collectively known as tauopathies (Kovacs et al. 2022). In AD, the commonest type of tauopathy, abnormally phosphorylated tau (pTau) accumulates in form of NFTs, dystrophic neurites and neuropil threads. The importance of tau dysfunction in AD is underscored by the fact that the degree of cognitive impairment and dementia correlates strictly with the amount of tau burden in AD brain (Creekmore et al. 2024b). Given the failure of all currently available pharmaceutical trials aimed at reducing the amount of Aβ in AD patients, elucidating the role and mechanism of tau accumulation becomes essential for developing alternative and effective therapeutic strategies; studies of pTau at the ultrastructural level are therefore of the outmost importance.

In AD, tau undergoes abnormal phosphorylation and as a consequence detaches from microtubules, aggregates into paired helical filaments (PHFs) and straight filaments (SFs) and accumulates in form of NFTs (Ikeda et al. 1992). The twisted nature of the aggregates was first shown at the ultrastructural level in 1963 by Kidd (Kidd 1963). Early studies using conventional TEM detected ~ 15 nm thick straight and twisted tubules within neurons, degenerating neurites and in the extracellular “ghost” tangles (Crowther 2021). Subsequent studies using immunogold techniques at the ultrastructural level helped identifying tau as a protein associated to microtubules within normal tissue (Migheli et al. 1988), as well as the main component of PHFs and SFs in AD brain in its abnormally phosphorylated form (Crowther and Goedert 2000). Soon it was shown by immunogold EM that pTau localizes not only to NFTs, but also to ribosomal and periribosomal granular regions of neurons, suggesting a role of pTau in early impairment of the translational machinery (Papasozomenos 1989).

#### Recent Advances

Ultrastructural evidence of the association to ribosomes has been recently confirmed with a panel of antibodies to multiple pTau epitopes, which revealed that pTau association in granular pretangles and perinuclear zones precedes the appearance of more mature fibrils, thus implying mislocalization of tau as an early sign of neuronal dysfunction (Price et al. 2025). By using advanced techniques such as quantum dot labeling for correlating LM and EM, it was shown that LM-defined pretangles are actually tau-positive SFs appearing before classical NFTs become recognizable, and that the AD pretangles have a distinct appearance from those of other tauopathies (Tatsumi et al. 2014).

Recently, the development of cryo-EM techniques at a near-atomic resolution has provided a tremendous advancement in clarifying the formation and the role of pTau aggregates in AD (further details are available in Table 3). By mapping PHFs and SFs at 3.4–3.5 Angstrom resolution, Fitzpatrick et al. (Fitzpatrick et al. 2017) first showed that filament cores are made of two identical protofilaments comprising residues 306–378 of tau protein, which fold into a cross-β/β-helix structure, and that PHFs and SFs differ in their protofilament packing, definitely demonstrating ultrastructural polymorphism of the tau aggregates. Subsequent cryo-EM studies have further mapped the tau fold, showing that it differs from those described in other tauopathies, and identifying an AD core region of 79 aminoacids with five β-strands, which gives rise to two antiparallel β-sheets plus a β-helix region that also serves as a target for high-affinitiy PET ligands (Shi et al. 2021). Interestingly, cryo-EM studies in Down syndrome (DS), a condition with increased risk of developing early onset AD, have revealed PHFs and SFs made of C-shaped protofilaments with the same cross-β/β-helix structure of AD, underscoring the ultrastructural similarity at the molecular level of tau pathology across AD-related diseases (Ghosh et al. 2024).

**Table 3:** Cryo-EM contribution to the understanding of tau structure and filament formation: this table summarizes key structural findings regarding tau filaments with the use of Cryo-EM alone or in combination with other techniques

Conformation	Synthetic/Ex vivo	Technique	References
Two identical protofilaments, comprising residues 306–378 of tau protein, adopt a combined cross-β/β-helix structure and define tau aggregation, forming PHFs and SFs	Human AD cortex extracts	Cryo-EM	Fitzpatrick et al. (2017)
PHFs outnumbered SFs in all AD cases. Two C-shaped protofilaments with a combined cross-β/β-helix structure. No significant variation in tau filament structures between different individuals with AD	PHF and SF from SAD and FAD	Cryo-EM, immunogold NS-TEM	Falcon et al. (2018)
RNA is necessary for fibril integrity and binds to fibril surface on Arg406 and His407	Recombinant full-length tau in the presence of RNA	Cryo-EM	Abskharon et al. (2022)
The study reports in vitro assembly conditions that recapitulate AD and chronic traumatic encephalopathy filaments	In vitro assembly of recombinant tau	Cryo-EM	Lövestam et al. (2022)
Tau filaments (with human mutant P301S Tau) from Tg2541 and PS19 mice have a different filament core and differ from wild-type tau filaments from human brains	Tg2541 and PS19 mice brain extracts	Cryo-EM	Schweighauser et al. (2023)
Following seeding with the sarkosyl-insoluble fraction from AD brain, abundant filaments of HA-1N3R tau with a single protofilament were present that closely resembled the AD tau fold	SH-SY5Y cells that transiently expressed N-terminally HA-tagged 1N3R or 1N4R human tau, using brain extracts from individuals with AD or corticobasal degeneration	Immunogold EM, Cryo-EM	Tarutani et al. (2023)
PHF and SF are identical to those of AD cases	Human DS cortex extracts	Cryo-EM	Ghosh et al. (2024); Fernandez et al. (2024)
Tau clusters are arranged in parallel. Clusters are both inside cells and extracellular. Each cluster was composed of different ensembles of tau filaments (PHF only, SF only, or a combination of PHF and SF)	In situ, human post-mortem AD brains	Cryo-CLEM, Cryo-ET, cryo-FIB-SEM	Gilbert et al. (2024)
AD tau core fold was reconstituted while the proportion of PHFs was highly variable	Recombinant Tau^297−391^ in vitro filament formation	NS-TEM, Cryo-EM	Glynn et al. (2024)
Tau forms a diverse array of pore-like oligomers composed by two layers	AD brain extracts	Negative staining TEM, Cryo-EM	Dasari et al. (2025)
Phosphorylations in unstructured fuzzy coat region are essential for full seeding competence	Recombinant tau filaments (PAD12 tau) and extracted from AD brain	Cryo-EM, NS-TEM	Kasen et al. (2025)
Both mutants seeded further aggregation, while the wild-type formed maily unfolded aggregated; the C322A variant formed filaments partially resembling the AD PHF structure	Recombinant 0N3R tau forms (the wild-type and the C322A and C322S variants)	Cryo-EM	Santambrogio et al. (2025)

Moreover, templated seeding studies in SH-SY5Y cells seeded with extracts derived from AD and other tauopathies have shown by cryo-EM some degree of templating, suggesting that tau filaments in vitro resemble disease-specific conformations (Tarutani et al. 2023), thus supporting a prion-like model of propagation of toxic tau (Dujardin and Hyman 2019). The latter hypothesis is also supported by findings of an association of assembled tau with EVs (Ruan et al. 2021). A better understanding of the molecular mechanism underlying EV-mediated secretion of tau has been provided by recent cryo-ET and single-particle cryo-EM studies, that revealed truncated tau contained within EVs enriched in endo-lysosomal proteins, along with interactions between tau filaments and molecules that anchor them to the EV limiting membrane, suggesting a process of selective packaging (Fowler et al. 2025).

Attempts to assembly in vitro recombinant tau into filaments that are structurally identical to PHFs of AD have been successfully shown by cryo-EM analysis (Lövestam et al. 2022). These studies are of the greatest importance, as in vitro reconstructed tau filaments may clarify the relationship between ultrastructure, aggregation/folding kinetics, phosphorylation dynamics, thus opening new avenues for developing chemical compounds which may interfere with phosphorylation and fibril formation (Huseby and Kuret 2016).

Another powerful application of ultrastructural EM techniques has been directed to understanding the assembly of soluble tau oligomers purified from AD brains. Negative-staining TEM and atomic force microscopy (AFM) showed that pTau assembles into misfolded highly heterogeneous oligomers. The bigger oligomers with a diameter of 10–20 nm were observed in Cryo-EM (with a 2.5–4 Å resolution), which revealed ring-like structures with two thin layers separated by approximately 1 nm; this structure could enhance their interactions with cellular membranes and proteins (Dasari et al. 2025).

Comparative ultrastructural studies have allowed to analyze and classify tau polymorphisms across the various tauopathies: cryo-EM and NS-TEM analyses can differentiate AD from Pick disease, progressive supranuclear palsy and corticobasal degeneration, based on fold length, β-helix structure and protofilament organization, supporting the strain propagation model of distinct toxic tau conformations (Creekmore et al. 2021, 2024b; Goedert 2021; Lövestam et al. 2022).

While TEM and cryo-EM have generated an impressive amount of data regarding the formation and assembly of pTau subcellular structures in AD, SEM techniques have been fundamental for elucidating the three-dimensional structure of NFTs, revealing tau positive filamentous assemblies in situ with nanometer scale resolution. In a seminal paper using freeze cracked human autopsy tissue, both PHFs and SFs were readily distinguished, the first with a diameter of 28 nm and periodic constrictions of 100 nm, the second with a diameter of 20–25 nm. In addition, atypical variants were observed, supporting a protofilament model of PHFs (Itoh et al. 1997).

On the other hand, scanning TEM (STEM) has been employed to directly determine the mass-per-length (MPL) of individual tau fibrils isolated from AD brains, showing that a single polypeptide can give rise to both polymorphic fibrils and diverse globular oligomers simultaneously (Goldsbury et al. 2011). Using cryo-ET, combined with cryo-FIB-SEM lift-out to prepare tissue lamellae, tau inclusions were observed forming parallel arrays of unbranched filaments. Subtomogram averaging of 136 tau filaments within a single tomogram allowed determination of the polypeptide backbone conformation and the filament polarity of paired helical filaments (PHFs) in tissue (Gilbert et al. 2024) (Fig. 1B).

Using SEM, energy dispersive spectroscopy (EDS) and electron energy loss spectroscopy (EELS), pTau deposits were shown to colocalize with iron and zinc deposits in AD brain, suggesting a link between tau pathology and oxidative metal deposition (Madsen et al. 2020).

Finally, the recent advances in vCLEM and the availability of large datasets have allowed the identification of an unusual localization of pTau, i.e. inside spine-like protrusions at the axon initial segment. These structures could be prone to detachment and, therefore, might be involved in the formation of exosomes—a process implicated in the dissemination of pTau in AD (Han et al. 2023).

In summary, SEM techniques have provided useful data to elucidate not only the morphology of tau aggregates (bundle dimensions, filament diameter, periodicity), but also the spatial localization within neurons and the pathological microenvironment of NFTs, thus complementing the traditional investigation of tau with TEM and cryo-EM.

As a final word, data from ultrastructural studies have also been exploited to develop mathematical models of tau propagation across brain regions. Using data from imaging and ultrastructural staging studies, these theoretical models can predict region-specific spreading patterns of tau, not only by diffusion, but also by axonal transport, consistent with the clinical progression (Tora et al. 2025).

### Synapses

#### Background

Synaptic degeneration is already evident in AD early-stages, preceding neuronal loss, and is one of the earliest and most predictive features of AD (Meftah and Gan 2023). Accordingly, synaptic loss functionally correlates with cognitive impairment in AD (de Wilde et al. 2016a, 2016b).

Axons and dendrites have long been recognized as key components in AD pathology. Terry et al. (1964) observed that, in the periphery of plaques, these structures appeared enlarged and contained abnormal neurofilaments and, occasionally, dense bodies (Terry et al. 1964). Similarly, Krigman in 1965 and later Gonatas (Gonatas et al. 1967) reported enlarged presynaptic endings that also contained dense bodies.

This concept gained quantitative support over the following decades. In 1990, DeKosky et al. demonstrated an important reduction in synapse density in the frontal cortex of AD patients. Accordingly, there was a significant correlation between synapse count and Mini-Mental State examination scores (DeKosky and Scheff 1990). The synaptic degeneration was further characterized by immunogold EM labeling, thus reinforcing the hypothesis of progressive synaptic pathology in AD neocortex (Masliah et al. 1991).

Taken together, these foundational studies laid the groundwork for our current understanding of AD as a synaptopathy and established EM as a crucial tool for exploring the disease’s earliest microstructural changes.

#### Recent Advances

In recent years, the advent of advanced vEM techniques, such as FIB-SEM, has allowed the micro-anatomical characterization of the neuropil of brain regions involved in AD, revealing region-specific synaptic loss. It was thus shown that while transentorhinal cortex thickness is reduced in AD, leading to a general loss of overall synapse number, the synaptic density is maintained (Domínguez-Álvaro et al. 2018). In this area, asymmetric synapses, that are classified as excitatory based on their post-synaptic density (Peters and Palay 1996), were more fragmented than in controls (Domínguez-Álvaro et al. 2019). Likewise, when the same technique was applied to the CA1 area of the hippocampus, early cases of AD did not display changes in synapse density, nor in excitatory/inhibitory synapse ratio, although there was already evidence of cortical atrophy and of changes in the distribution of postsynaptic targets and synaptic shapes. On the other hand, late AD cases suffered a decrease in synaptic density and morphological alterations of the remaining synapses (Montero-Crespo et al. 2021).

Additionally, TEM with serial sectioning of bioptical samples of AD frontal cortex revealed that a reduced synapse density appears to coincide with both a rise in the number of short spines and an enlargement of spine necks, supporting the view that structural spine changes could be implicated in the initial stages of cognitive impairment in AD (Androuin et al. 2018).

Interestingly, as an alteration of the olfactory areas is observed in almost 90% of AD patients, these regions were also shown by TEM to display Aβ accumulation, accompanied by distorted postsynaptic densities and a decline in presynaptic vesicles (Son et al. 2022).

EM techniques have also allowed a better understanding of synaptic loss progression, showing an increased reduction of synapses near the plaque border, a region specifically enriched in Aβ oligomers, as shown by immunogold labeling. This effect was proportional to plaque size in both APP-PS1 mice and human AD hippocampus. In the presynaptic element, synaptic vesicles were reduced, while autophagic vacuoles were increased (Sanchez-Varo et al. 2021) (Fig. 1C).

Abnormalities in neurotransmitter expression, that coexist with synaptic dysfunction in AD, have been also investigated and clarified at the ultrastructural level. For example, in APP-PS1 mice, immunogold EM revealed mislocalization of GABAB1 receptors in the CA1 region, with an increased presence in the cytoplasmic compartment (Martín‐Belmonte et al. 2020a, 2020b, 2020c; Martín-Belmonte et al. 2020a). In the same model, SDS-FRL demonstrated a reduction of AMPA and mGlu5 receptors at synaptic sites (Martín-Belmonte et al. 2021, 2020b). Notably, SDS-FRL also indicated that pTau accumulation contributes to receptor mislocalization (Alfaro-Ruiz et al. 2022).

Conversely, in the APP^NL−F^ mouse model, no change in GABA_A_ receptor positivity and size of somatic and dendritic synapses of hippocampal interneurons was observed via correlative LM and SBF-SEM, suggesting a neuron-specific vulnerability/resilience to amyloidosis (Sos et al. 2020).

Mitochondrial dysfunction could be another key mechanism underlying synaptic loss in AD, as shown by EM. SBF-SEM revealed region-specific reductions in mitochondria within pre- and postsynaptic compartments in APP-PS1 and 5xFAD mice (Seo et al. 2021). Similarly, TEM in human BA41/42 and BA46 cortex showed fewer presynaptic terminals with multiple mitochondria in AD, along with abnormal mitochondrial shapes, synaptic multivesicular bodies, and shorter synapse apposition near plaques (Pickett et al. 2018).

Finally, immunogold EM and array tomography also revealed oligomeric Aβ species inside the synaptic terminals of plaque-bearing AD mice (Pickett et al. 2016). In this context, large vEM datasets may help understanding the interplay between classical AD hallmarks (Aβ and NFTs) and region specific synaptic alterations (Han et al. 2023).

These findings confirm that synaptic degeneration is a central event in AD and that EM is the gold standard for visualizing synaptic loss, receptor mislocalization, organelle damage and plaque interactions—reinforcing the view of AD as a synaptopathy.

### Mitochondria

#### Background

Mitochondria are key regulators of cellular energy and homeostasis, supporting ATP production, redox balance, calcium buffering and play a balancing role in the apoptotic process (Wang et al. 2020). In AD, glucose hypometabolism and altered mitochondrial enzyme activity reveal marked mitochondrial dysfunction, leading to oxidative stress, calcium imbalance, and structural abnormalities that promote neuronal loss and neurodegeneration (Readnower et al. 2011). Notably, both Aβ and hyperphosphorylated tau can directly impair mitochondrial function, potentially establishing a self-perpetuating cycle of cellular damage Zhang et al. 2024a).

In 1985 Saraiva et al. first demonstrated mitochondrial alterations at the EM level in apparently normal dendrites of AD frontal cortex. The alterations included increased matrix density and paracrystalline inclusions within the intercristal space. Later, in 2001 Velez-Pardo et al. found via EM swollen mitochondria in TUNEL-positive brain slices of frontal cortex and hippocampus of terminal PS1E280A AD brains, also showing that necrosis is the most frequent form of cell death in this context (Velez-Pardo et al. 2001).

#### Recent Advances

Recently, EM study in AD cortex confirmed mitochondrial impairment both at the pre- and post-synaptic compartment (Wang et al. 2023).

Over the past decade, the development of various AD mouse models has enabled more detailed and systematic investigations into mitochondrial abnormalities.

Concerning mitochondrial dynamics, diverse alterations were found in different animal models for AD. For instance, TEM data revealed pronounced mitochondrial fragmentation and reduced mitochondrial length in both hippocampal and cortical tissues of seven-month-old hAbKI mice (Kshirsagar et al. 2022) and in APP-PS1 mice starting at 3 months of age (Xu et al. 2017). Importantly, these alterations appear to be influenced not only by Aβ accumulation but also by tau pathology, suggesting that both proteins contribute to impaired mitochondrial dynamics. Indeed, TEM analysis of the hippocampus of 12-month-old mice bearing the P301L tau mutation revealed significantly increased mitochondrial number and reduced mitochondrial length, indicating increased fission and reduced fusion, and suggesting that hippocampal accumulation of phosphorylated tau is responsible for the abnormal dynamics of mitochondria (Kandimalla et al. 2018).

An interesting finding was discovered in 2016 by Zhang et al., using vEM reconstruction in the brain of both patients and of five mouse models of AD. They described a new fission arrest mitochondrial phenotype, defined as "mitochondria-on-a-string" (MOAS), that results in elongated teardrop-shaped organelles (0.5 μm in diameter), connected by thin double membranes extending up to 5 μm long. This morphology may occur at the final stages of fission process as a response to energetic stress (Zhang et al. 2016) (Fig. 1D). The same phenotype was observed in dendrites of dorsolateral prefrontal cortex neurons from aged rhesus macaques (Morozov et al. 2017) and in the OXYS SAD mimicking rat model, where the MOAS phenotype was significantly expressed, especially in pre-symptomatic stages (Tyumentsev et al. 2018). Altogether, the evidence suggests that mitochondrial dynamics may have a major role in regulating neuronal survival, particularly in the early phases of AD.

EM techniques have also substantially increased our knowledge of the regional and cellular compartment specificities of mitochondrial damage and loss. Accordingly, using SBF-SEM in 5xFAD mice, specific loss of pre-synaptic mitochondria was found in the medial prefrontal cortex, but not in the primary visual cortex (Seo et al. 2021). Moreover, in cortical biopsies (parietal, prefrontal or frontal cortex) from AD patients, the number of pre-synaptic mitochondria was significantly reduced, along with a significantly increased damage to mitochondria; these data correlated with changes in synaptic vesicle density. Mitochondria in the post-synaptic dendritic spines were also enlarged and damaged in AD biopsies (Pickett et al. 2018).

In recent years, EM has helped clarifying the interaction between mitochondria and other structures/organelles affected in AD. A few instances will be presented here.

Accumulation of Aβ inside mitochondria has been observed in mAPP-HT22 cells, following accumulation of mutant APP (mAPP) and Aβ (Reddy et al. 2018). In nanogold labeled yeast mitochondria, it was shown that Aβ enters the mitochondria following rcognition by the TOMM20 receptor (Hu et al. 2018). Conversely, a recent CLEM and cryo-EM study showed that Aβ42 aggregates do not interact with mitochondria, although in some instances they are closely located, suggesting that other mechanisms should be considered as the source of mitochondrial dysfunction. At the same time, the study proposes cryo-EM as a gold-standard technique for high-resolution visualization of the mitochondrial membranes and internalized proteins, such as the OXPHOS system proteins, that could be altered in AD (Nesterov et al. 2024).

In APP-PS1 mice injected with adeno-associated virus encoding the mutant human tau P301L, tau immunoparticles were observed in dendrites and accumulated as aggregates within mitochondria of APP-PS1 mice, whereas no such accumulation occurred in wild-type (wt) animals, indicating a potential mechanism underlying synaptic dysfunction (Cuadrado-Tejedor et al. 2021). The same interaction between tau and mitochondria is present in mice expressing the P301L mutation (Kandimalla et al. 2018).

Moreover, EM has provided conflicting results both in cell lines and animal models, regarding the effect of FAD related mutations on ER-mitochondria contacts (Del Prete et al. 2016). More thorough studies, e.g. using vEM techniques, are greatly needed to clarify this aspect.

Finally, mitochondrial alterations have also been found in a model that relate stress to AD trajectories. More in detail, early-life stress seems to aggravate mitochondrial loss at the pre-synapse in APP-PS1 mice (Kotah et al. 2024); similar effects were observed in 3xTgAD following sleep fragmentation (Liu et al. 2025). These results highlight the importance of further EM studies on mitochondrial alterations and dynamics in SAD, where environmental factors may play a major role (Suresh et al. 2023).

In the future, integration of different EM techniques, such as cryo-EM and CLEM, will likely dissect the dynamics and the spatial relationship between mitochondria and tau, Aβ, ER contacts and other organelles affected in AD. This will strengthen the use of mitochondrial ultrastructure as a robust AD early biomarker and therapeutic target, both in familial and sporadic form of AD, even though the precise nature of the relationship between mitochondrial impairment and protein aggregation is at present unclear. High resolution studies are much needed to clarify whether impaired proteostasis stems from mitochondrial impairment, or whether the latter is a consequence of increased protein aggregation.

### Iron Deposits

#### Background

Iron, the most abundant transition metal in the body, is essential for oxygen transport, energy production and enzymatic activity. However, its redox properties also make it potentially harmful, as it can generate reactive radicals, damaging cellular components. Lacking a regulated excretion pathway, iron accumulates with age—particularly in brain regions vulnerable to neurodegeneration, potentially leading to oxidative stress, inflammation, and cell death (Mezzanotte et al. 2024). Notably, ferroptosis, an iron-dependent form of cell death, has been implicated in neurodegeneration, including AD (as reviewed by Stockwell et al. 2017; Yan and Zhang 2020).

The potential connection between iron accumulation and AD was first noted by Goodman in 1953 (Goodman 1953). Later, in 2004, electron nanodiffraction and high-resolution transmission electron microscopy (HR-TEM) enabled detailed characterization of pathological ferritin associated with tau filaments in the Alzheimer’s brain (Quintana et al. 2004), revealing the presence of a hemosiderin component.

Following this work, in 2006, analytical TEM and immunogold labeling localized ferritin together with hemosiderin at the periphery of senile plaques, inside sulfur-rich dense bodies of dystrophic neurites. Hemosiderin was also found in lysosomes and siderosomes of glial cells (Quintana et al. 2006).

Later, in 2008 Collingwood et al. (Collingwood et al. 2008) used a three-dimensional tomographic imaging combined approach to characterize the iron-rich plaque core material, identifying biogenic magnetite and/or maghemite as the dominant iron compound. This finding suggested that abnormal iron biomineralization processes could play a role in aberrant protein aggregation.

#### Recent Advances

Recent studies using an integrated set of advanced TEM techniques have demonstrated that iron is present in the cores of amyloid plaques as iron oxide (Fe₃O₄) magnetite nanoparticles, directly incorporated into fibrillar Aβ, while copper and zinc are distributed along the amyloid fibers (Plascencia-Villa et al. 2016) (Fig. 1E). Interestingly, the magnetite nanoparticles found in human brains closely resemble high-temperature magnetite nanospheres produced by combustion or frictional heating, both of which are common components of urban airborne particulate matter (PM). With diameters below 200 nm, these particles are capable of directly accessing the brain via the olfactory nerve, particularly through a compromised olfactory system (Maher et al. 2016), or by endocytosis through endothelial cells, as shown also in vitro through TEM (Gárate-Vélez et al. 2020). Thus, environmental and/or endogenous iron could be considered as a possible risk factor for AD.

Furthermore, the capacity of amyloid peptide Aβ42 to bind and concentrate iron hydroxides, promoting magnetite formation, was demonstrated by a combination of different EM techniques (Tahirbegi et al. 2016).

The bi-directional relationship between Aβ and iron was further studied by Everett et al. (Everett et al. 2020) who, by using EM and X-ray spectromicroscopy, showed that when Aβ and iron co-aggregate, Aβ in turn is responsible for the conversion of the inert ferric core of ferritin into more reactive low-oxidation-states.

Moreover, metals can also interact with tau. TEM studies of tau aggregates formed in the presence of metal ions suggest that the latter may influence the aggregation process and that phosphorylation of the full-length htau40 reduces metal interactions (Ahmadi et al. 2019).

At the subcellular level, SEM coupled to energy-dispersive X-ray spectroscopy showed that iron and aluminum colocalize in the neuronal nuclei of AD brains (hippocampus and temporal lobe) in both hetero- and euchromatin, and are particularly concentrated in the nucleolus (Yumoto et al. 2018).

Furthermore, combined methodologies were applied to map and analyze iron deposition and its oxidation states in AD, utilizing human brain autopsy samples from individuals with advanced AD who had previously been examined through correlative MRI-histology. Through SEM, these regions were found to be iron-rich, while using 3D with FIB-SEM the deposits appeared to contain iron and occasionally zinc, overlapping with amyloid and tau deposits. On the other hand, STEM combined with energy loss spectroscopy (STEM-EELS) allowed to discriminate the relative iron oxidation state (Zeng et al. 2021).

Finally, in a recent work, the frontal cortex of middle-aged APP-PS1 and wt mice was stained with Pearl’s DAB and analyzed with SEM. Compared to wt mice, APP-PS1 mice exhibited higher iron accumulation and clustering, with parenchymal iron signals localized to oligodendrocytes, pericytes, astrocytes, microglia, infiltrating macrophages, and amyloid plaques. Interestingly, both wt and APP-PS1 mice showed an increased number of iron-rich cells at the CNS interface and within perivascular spaces. Ultrastructural analysis further revealed numerous cells containing secretory granules loaded with iron (Lau et al. 2025). These findings reinforce that AD is associated with elevated iron deposition and indicate that the recently identified iron-rich cells along the CNS border may also contribute to iron accumulation during normal aging.

Interestingly, iron chelators—designed to reduce iron overload—represent a potential therapeutic strategy for AD (Gleason and Bush 2021). Although current interventional trials have not yet demonstrated significant success, ultrastructural observations suggest that this approach warrants further investigation (Mandal et al. 2024).

### Blood–Brain Barrier

#### Background

The blood–brain barrier (BBB) is a continuous tight endothelial membrane that prevents the entrance of many circulating factors into the CNS. Its breakdown facilitates the entrance of neurotoxic blood derivates, cells and microbes, causing inflammatory and immune responses. Importantly, BBB dysfunction has been observed as an early sign of AD before the onset of dementia or neurodegeneration, with the ApoEε4 allele representing a key genetic risk factor for BBB impairment (Sweeney et al. 2018b).

The first ultrastructural evidences date back to 1979, when Miyakawa et al. Miyakawa and Uehara highlighted how amyloid and degenerated blood vessels in the central core of senile plaques were in close relation and, conversely, blood vessels with amyloid angiopathy were surrounded by amyloid deposits.

Alterations of the capillary bed were further described through EM in subsequent years. A significant thickening of the basement membrane was observed (Mancardi et al. 1980). Endothelial cells were in a degenerated state and surrounding astrocytic feet appeared swollen (Higuchi et al. 1987). Cerebral vessels were irregular, covered by rounded or conical extrusions, lacking the perivascular plexus of nerve fibers and often displaying perforation changes; infiltration of the vascular wall with rounded cell-like bodies, sometimes resembling pericytes or monocytes, was observed (Scheibel et al. 1987). Scheibel suggested the occurrence of a denervating microangiopathy, caused by early degeneration of the locus coeruleus and nucleus basalis of Meynert (Scheibel 1987). Conversely, both content and morphology of mitochondria were unchanged (Mancardi et al. 1985).

#### Recent Advances

During the last 10 years, the bidirectional interplay between amyloid and brain vessels was further investigated. In a mouse model of chronic cerebral hypoperfusion (CCH), it was shown via TEM that CCH facilitates both the intracellular and extracellular deposition of Aβ (Wang et al. 2016).

Moreover, a deeper understanding of the timing of microvascular pathology was achieved thanks to the APP-PS1 mouse model. By SEM imaging, Kelly et al. found that 4 to 5 month-old APP-PS1 mice, in the absence of cognitive impairment, display microvascular alterations, not only in the brain but also in liver, spleen and kidney (Kelly et al. 2017) (Fig. 1F). In the same mouse model, following ischemia-hypoperfusion, TEM revealed increasing numbers of empty mitochondria in hippocampal neurons and proliferation of blood-vessel walls (Sun et al. 2022).

Finally, BBB alterations were confirmed through EM in alternative animal models of AD, such as Slit-2 transgenic mice (Li et al. 2014) and APP knock-in zebrafish (Pu et al. 2017).

Altogether, these findings underscore the value of EM techniques to study the BBB, not only for validating the vascular damage, but also in supporting the emerging view of BBB dysfunction as a key and possibly initiating event in Alzheimer disease (Alkhalifa et al. 2023).

### Autophagy

#### Background

Autophagy, first described in 1960 by Christian de Duve (Harnett et al. 2017), is a catabolic process responsible for degrading and recycling damaged proteins and organelles, that has emerged as a key player in preserving cellular homeostasis.

In AD, the autophagic flux is frequently impaired, leading to accumulation of autophagic vacuoles and partially degraded substrates within neurons (Sayed et al. 2024).

In 1967, Suzuki and Terry first observed numerous aberrant subcellular vesicles together with PHFs within dystrophic neurites in the brain of AD patients (Suzuki and Terry 1967). On the other hand, dystrophic neurites are a common appearance in other cerebral amyloidopathies as well and, in pioneering immunogold EM studies on prion diseases, vesicles and dense bodies of dystrophic neurites surrounding amyloid plaques were clearly labeled by ubiquitin, a small heat shock protein involved in autophagy and proteasomal degradation (Migheli et al. 1991). In 2005, Nixon and colleagues, using immunogold EM for cathepsin D and calnexin on neocortical biopsies from AD brain, identified these vesicles as autophagosomes and other prelysosomal autophagic vacuoles (AVs). AVs were more abundant in AD brains compared to controls, particularly within neuritic processes, including synaptic terminals (Nixon et al. 2005).

#### Recent advances

In the last ten years, autophagic flux alterations have been observed by TEM in different cell models: primary and immortalized hippocampal neurons overexpressing human full-length wt tau or mAPP and Aβ demonstrated an impairment in autophagy following aberrant protein accumulation Reddy et al. 2018). Moreover, in primary neurons from AD transgenic mice and wt neurons treated with exogenous Aβ, MVBs appeared enlarged compared to wt neurons (Willén et al. 2017). Interestingly, *N*-terminally pyroglutamylated Aβ3(pE)-42 (a significant constituent of amyloid plaques), but not Aβ1-42, was found by immunoEM using DAB to accumulate and disrupt the endolysosomal system of primary murine astrocytes (Wirth et al. 2024).

Conversely, Lee et al. recently demonstrated by CLEM approach that in various AD mouse models a decline in the acidification of neuronal autolysosomes precedes extracellular amyloid deposition. Notably, a particular pattern of neurodegeneration, called PANTHOS -meaning poisonous anthos (Greek for flower)- was described in compromised but otherwise intact neurons, via TEM and SBF-SEM. Profuse Aβ-positive AVs appeared packed into large membrane blebs forming flower-like perikaryal rosettes. It was suggested that failure of an initially protective autophagic response in neurons is accompanied by PANTHOS occurrence, leading to neuronal cell death. Notably, this phenotype was also recently confirmed in AD biopsies (Lee et al. 2022) (Fig. 1G).

Interestingly, dysregulation of mitophagy, the process of damaged mitochondria clearance, has been implicated as a contributor to AD pathology (Zhang et al. 2024b) and is also reported in other neurodegenerative diseases (Cai and Jeong 2020). In this context EM has been instrumental in showing that aged APP-PS1 mice with high mitochondrial damage exhibited enhanced mitophagy and impaired mitochondrial clearance, leading to autophagosome accumulation and metabolic abnormalities (Li et al. 2023).

Moreover, it was shown through TEM that an enhancement in astrocytic autophagy improves Aβ clearance, both in primary cortical astrocytes and in APP-PS1 mice (Kim et al. 2024).

Using immunogold EM, Aβ42 fibrils were found within neuronal lysosomes in APPxPS1-KI mice. The organelles appeared enlarged and contained dense material as well. In AD patients, instead, an accumulation of undigested material was present in lipofuscin granules in neurons, however this material was not Aβ-immunoreactive (Brainbank NeuroCEB Neuropathology Network et al. 2022).

Moreover, recent EM studies characterized the granulovacuolar bodies (GVBs) as membrane-bound vacuolar structures with a dense core, that accumulate in the brain of patients with various types of neurodegenerative disorders (Wiersma et al. 2019). Wiersma and colleagues first demonstrated that seeding of tau pathology triggers the formation of GVBs in different mouse models in vivo and in primary mouse neurons in vitro (Wiersma et al. 2019). It was subsequently shown that structurally and morphologically different tau assemblies can diversely affect GVB accumulation in primary neurons (Jorge-Oliva et al. 2023).

Notably, these alterations seem to follow a sex- and region-specific pattern. For instance, Adlimoghadd et al. showed in the 3xTg-AD model a significant increase of the mitophagosome number in the cortex of affected males compared to controls, while in the female the increase was significant both in the cortex and the hippocampus, and correlated with cognitive deficits (Adlimoghaddam et al. 2025).

Finally, new models suitable to study the autophagic flux are being developed, e.g. *Drosophila white;yata* mutants and *white* mutants, which express in the eye a mutation that affects the trafficking of an APP-like protein; in this model, EM allowed to observe abnormal bleb-like structures containing MVBs and autophagosomes (Arimoto et al. 2019).

Although the exact role of autophagy in AD neurite degeneration is still under investigation, thorough EM studies have shown that adding rapamycin, an inducer of autophagy, to PC 12 cells treated with Aβ25-35 peptide efficiently suppresses neuritic degeneration (Yang et al. 2014). This finding opens new potential avenues for treating AD patients with drugs that upregulate autophagy (Kaeberlein and Galvan 2019).

### Neuroinflammation

#### Background

Neuroinflammation encompasses inflammatory processes occurring within the central nervous system (CNS) and can be initiated by a variety of pathological stimuli, including infections, traumatic injury, ischemic events, or exposure to neurotoxic agents (Leng and Edison 2021). The innate immune response in the CNS is primarily coordinated by microglia and astrocytes. Additionally, capillary endothelial cells and infiltrating peripheral immune cells play a major role, particularly when the BBB is compromised, as reported in AD (Leng and Edison 2021).

In neurodegenerative disorders, neuroinflammation typically evolves into a chronic process that fails to resolve spontaneously and is increasingly recognized as a crucial driver of disease progression, if not of initiation itself (Leng and Edison 2021). Astrocytes, in particular, are known to react to various pathological stimuli—including mechanical injury, ischemia, and abnormal protein aggregates—by undergoing reactive gliosis, a hallmark of the neuroinflammatory response (Probst et al. 1982; Pekny et al. 2014).

The first ultrastructural evidence of a neuroinflammatory response in AD dates back to 1983, when Mancardi et al. performed an EM study of cortical biopsies of AD. They were able to reveal an increase in the dense astrocytic glia near the capillaries, compared to healthy, undemented individuals (Mancardi et al. 1983).

#### Recent Advances on Astrocytes

Recently, St Pierre and colleagues investigated the ultrastructural features related to alteration and heterogeneity of astrocytes in CA1 strata lacunosum-moleculare and radiatum in aged APP-PS1 mice. Astrocytes were found to interact heavily with synaptic elements and displayed increased phagolysosomal activity, compared to controls. Moreover, an electron-dense appearance of the astrocytes, hence dubbed as dark astrocytes, was identified, close to blood vessels; this phenotype was also observed in a human AD biopsy (St-Pierre et al. 2023).

As previously discussed, multiple ultrastructural observations show that astrocytes participate to synapse coverage (Kater et al. 2023) and iron accumulation (Lau et al. 2025) and are vulnerable to intracellular Aβ deposits (Konstantinidis et al. 2023). Besides being involved in AD neurodegeneration and contributing to neuroinflammation, astrocytes seem to have a role in propagating Aβ pathology. Human hiPSC-derived astrocytes were seen through TEM to truncate and pack Aβ in highly resistant aggregates (Beretta et al. 2024). In a co-culture of primary neurons and glial cells, astrocytes quickly internalized substantial amounts of Aβ protofibrils and released microvesicles containing N-terminally truncated Aβ, which triggered apoptosis in cortical neurons (Söllvander et al. 2016).

Interestingly, astrocytes seem to play a role in late onset AD by secreting ApoE, a protein involved in cholesterol metabolism and an important genetic risk factor for the disease. Primary astrocytes obtained from human *APOE* knock-in mice secrete ApoE as an antiparallel dimer, whose structure has been further characterized thanks to TEM and cryo-EM application (Strickland et al. 2024). In turn, the antiparallel conformation of ApoE is thought to impact on its binding to Aβ, influencing disease progression.

Finally, TEM images from APP-PS1 mice provide evidence of astrocytic phagocytosis of abnormal neuronal structures. Lysosomes were frequently observed in close proximity to engulfed dystrophic neurites, and immunogold labeling confirmed the presence of APP-positive and neuronal/synaptic components within the cytoplasm of phagocytic astrocytes. Astrocyte-mediated phagocytosis of pathological neuronal structures was also verified by TEM in human AD brain tissue (Gomez‐Arboledas et al. 2018).

#### Background

As for microglia, back in 1989 Itagaki et al. showed that reactive microglial cells were in intimate contact with amyloid fibrils, suggesting that amyloid deposition might precede microglial activation (Itagaki et al. 1989). In 1990 Perlmutter et al. studied the ultrastructure of microglial cells interdigitated with the amyloid fibrils: they had dark cytoplasm, dense bodies and distinctive nuclear appearance (Perlmutter et al. 1990).

#### Recent Advances on Microglia

In the last 10 years, new protocols have been published to investigate the interaction of microglia with Aβ plaques (Bisht et al. 2016a). Through SEM, microglia in 13-month-old male 5xFAD mice were observed in close contact with large Aβ aggregates, forming extracellular pockets directly adjacent to fibrillar material. These compartments stained positive for acid phosphatase, indicating the presence of “lysosomal synapses” at the microglia–Aβ interface (Jacquet et al. 2024). Furthermore, through correlative light, transmission, and scanning electron microscopy combined with array tomography (El Hajj et al. 2019), microglial cells near plaque regions in both an AD mouse model and late-stage AD patients were found to display impaired phagocytosis, dilated endoplasmic reticulum, small, elongated nuclei, increased lipid inclusions, and decreased IBA1 expression.

Moreover, C20 microglia treated with Aβ and imaged with SEM displayed different surface topographies, such as blebbed, pitted, or ruffled ones, and seemed to undergo amoeboid transition (Dyne et al. 2022).

Finally, a particular phenotype, called dark microglia (Bisht et al. 2016b), was better characterized thanks to EM analysis. Ultrastructural analysis characterized these cells by their condensed, electron-dense cytoplasm and nuclei, along with expression of multiple oxidative stress markers. In APP-PS1 mice, dark microglia showed increased interactions with dystrophic neurites compared to typical microglia. Nevertheless, both microglial types contained fibrillar Aβ in their cytoplasm when directly associated with an Aβ plaque. Notably, dark microglia located near plaques and dystrophic neurites engaged significantly less with non-dystrophic elements and blood vessels than typical microglia (St-Pierre et al. 2022) (Fig. 1H).

## Conclusions

We have presented a series of examples that illustrate the tremendous power of ultrastructural studies applied to AD. We have shown that, starting from traditional TEM/SEM analyses to the most advanced and sophisticated techniques at a near-atomic resolution, electron microscopy has been instrumental in allowing the accumulation of more and more data on the pathogenetic mechanisms of the disease. This increasing collection of data has not been limited to the two hallmarks of AD, namely Aβ and tau, but has also significantly expanded our understanding of other players involved in the degenerative process, namely neuroinflammation, mitochondria, synaptic pathology, autophagy, iron deposition and BBB impairment. Moreover, EM has empowered the possibility of comparing and correlating observations made in different settings, such as autopsy/biopsy samples from AD human brain, animal models, cell cultures and synthetic structures analogue to the insoluble proteins.

Future studies centered on EM techniques will likely be instrumental in devising compounds able to interfere with either oligomer or fibril formation, thus also clarifying the pathogenetic role of either structure. The novel information obtained with the aid of EM studies are—and will be even more in the future—essential to develop new molecules that, by interfering with the assembly and spreading of AD insoluble proteins and/or by stimulating clearance and other protective mechanisms, may be helpful in preventing, reversing or delaying the clinical course of this devastating disease. EM would be also fundamental for the characterization and use of laboratory model of AD, that will help unraveling the cause-effect and temporal relationship between different AD hallmarks, such as protein dishomeostasis, mitochondrial damage and autophagy impairment. In addition, as it is already occurring for other organs (see kidney (Abe and Ohno 2025)), volume EM could become a very useful tool for staging the evolution of disease in neuropathology and become a diagnostic tool for routine examination, allowing early diagnosis (Collinson et al. 2023; Kolotuev 2024). Indeed, there is substantial literature supporting the value of brain biopsies in the diagnostic work-up of dementia, particularly in early-onset cases. Such procedures may help address the diagnostic gap that persists despite clinical assessment, PET imaging, and cerebrospinal fluid biomarkers (Alaa Saadi Abbood et al. 2025).

## Data Availability

No datasets were generated or analysed during the current study.
